# Propagation of periodic director and flow patterns in a cholesteric liquid crystal under electroconvection

**DOI:** 10.1038/s41598-024-74551-w

**Published:** 2024-10-05

**Authors:** Jun Yoshioka, Hiroki Nobori, Koji Fukao, Fumito Araoka

**Affiliations:** 1 Department of Physical Sciences, Ritsumeikan University, 1-1-1 Noji-Higashi, Kusatsu, Shiga 525-8577 Japan; 2 RIKEN Center for Emergent Matter Science (CEMS), 2-1 Hirosawa, Wako, Saitama 351-0198 Japan

**Keywords:** Liquid crystals, Fluid dynamics

## Abstract

The electroconvection of liquid crystals is a typical example of a dissipative structure generated by complicated interactions between three factors: convective flow, structural deformation, and the migration of charge carriers. In this study, we found that the periodic structural deformation of a cholesteric liquid crystal propagates in space, like a wave, under an alternating-current electric field. The existence of convection and charge carriers was confirmed by flow-field measurements and dielectric relaxation spectroscopy. Given that the wave phenomenon results from electroconvection, we suggest a possible model for describing the mechanism of wave generation. The validity of the model was examined using the Onsager variational principle. Consequently, it was suggested that wave generation can be described by four effects: the electrostatic potential, mixing entropy, anisotropic friction due to charge migration, and viscous dissipation of the liquid crystal.

**Supplementary Information:**

The online version contains supplementary material available at 10.1038/s41598-024-74551-w.

## Introduction

When a flow is induced in a closed system, it typically circulates. As seen in the Rayleigh-Bénard convection, electroconvection and bioconvection, flow often forms a one- or two-dimensional periodic pattern, which is regarded as a dissipative structure^1–3^. Here, it should be noted that the flowing material is not necessarily a simple fluid; it can be a complex fluid, such as a liquid crystal (LC) or a living organism. In this situation, the flexible structures of these fluids interact strongly with the flow, which also forms the aforementioned structure. This complicated situation results in the appearance of various complicated phenomena^4–28^ similar to the fluid-structure interaction problem in fluid engineering^29–34^.

In the case of electroconvection in LC, convection results from the periodic accumulation of ionic impurities of charge carriers^2^. Under an electric field, accumulation occurs in a deformed director field with inhomogeneous electric conductivity, and convection is induced by the migration of impurities. The deformation of the director is attributed to the existence of dielectric anisotropy in the LC and the modulation of the electric field owing to the accumulation of charge; in addition to this, the convective flow stabilises the deformation. Consequently, a positive feedback loop is formed by three factors: the charge migration, convection, and director deformation; owing to this loop, the electroconvection is stabilised. In this standard model called “Carr-Helfrich instability,” we need to consider at least the above three factors interacting with each other. It has been reported that the interaction between the director and flow fields results in various complicated phenomena in the LC systems under potential gradient^35–39^. In addition to this fluid-structure interaction, the migration of the charge carriers should be analysed as they interact with both flow and structure under the existence of the electroconvection. Therefore, it is generally complicated and challenging to describe the mechanisms of the phenomena related to electroconvection, as seen in the generation of travelling waves including solitons^4–14^, bifurcation between turbulent states^15–18^, and the appearance of negative viscosity^19,20^.

**Fig. 1:**
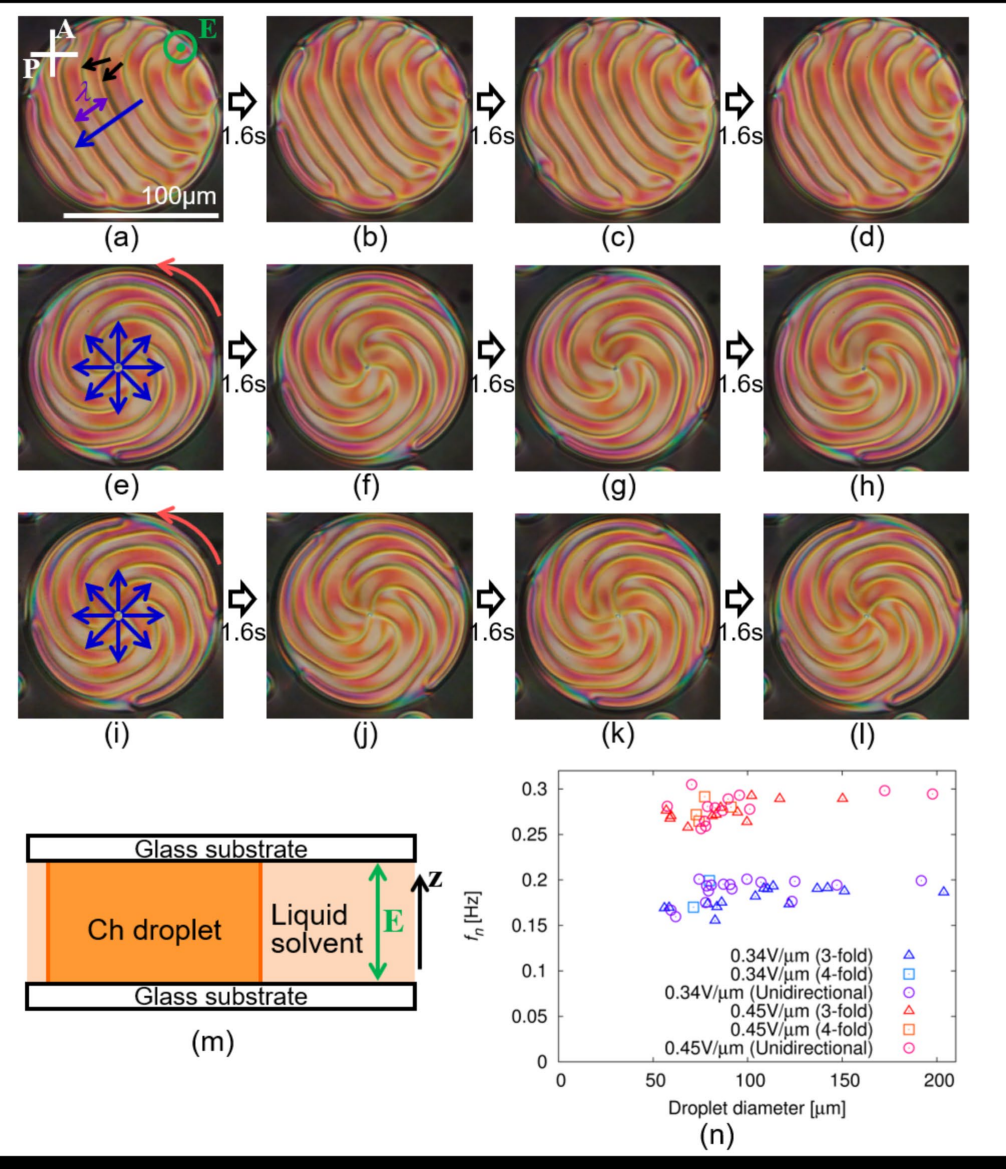
Propagation of fingerprint texture under an AC electric field. The corresponding movie is available as Supplementary Video 1. The concentration of the chiral dopant was 1.0 wt% and the cell thickness was 20 μm. (**a**–**l**) are POM images; P and A in (**a**) indicate polariser and analyser, respectively. (**m**) shows a schematic of the experimental system. An electric field **E** is applied along z-direction, as indicated by the green symbols in (**a**) and (**m**). The amplitude and frequency of the applied electric field were 0.34 V/µm and 160 Hz, respectively (*E*_0_ = 0.34 V/µm and *f* = 160 Hz). A sine wave was applied as the field (${E_z}={E_0}\cos (2\pi ft)$). The scale bar in (**a**) indicates 100 μm, and the time interval from (**a**–**d**), (**e**–**h**), and (**i**–**l**) was 1.6 s. In the fingerprint texture, the wavenumber *k* is defined as ${{2\pi } \mathord{\left/ {\vphantom {{2\pi } \lambda }} \right. \kern-0pt} \lambda }$, where *λ* is defined as indicated by the purple arrow in (**a**). In a period of the texture, two dark lines are observed as shown by the black arrows in (**a**). In (**a**–**d**), unidirectional propagation is observed in the direction of the blue arrow in (**a**). In (**e**–**h**) and (**i**–**l**), radial propagation is observed, as shown the blue arrows in (**e**) and (**i**). In these cases, the spiral texture with three- and four-fold symmetry was formed, showing a steady rotation as indicated by the red arrows in (**e**) and (**i**). (**n**) shows the dependence of the droplet diameter on the wave propagation frequency *f*_*n*_. The applied AC frequency *f* was 160 Hz, and the amplitude *E*_0_ was 0.34 V/µm for the symbols with cold colours and 0.45 V/µm for the warm-coloured symbols. *f*_*n*_ is constant under a constant *E*_0_ regardless of whether the propagation is unidirectional or radial.

In this study, we focused on electroconvection in a cholesteric (Ch) LC with a periodic structure owing to the spontaneous twisting of the director field. By applying an alternating-current (AC) electric field to the Ch LC, we found that the deformation of the director field propagated like a wave, as shown in Fig. 1 and Supplementary Video 1. Considering the discussion thus far, we also analysed the flow of the LC and the migration of the charge carriers to describe the mechanism of the wave propagation phenomenon in the director field. The flow field was measured using the fluorescence photobleaching method^25,40^, and to estimate the charge migration speed, the relaxation time of the electrode polarisation process was measured using dielectric relaxation spectroscopy (DRS)^41,42^. Based on the experimental results, a simplified model of the present situation was designed. By applying the Onsager variational principle^43^ to the model, we provided a possible description of the wave propagation phenomenon in Ch LC under electroconvection.

## Results

### Wave generation under AC electric field

**Fig. 2:**
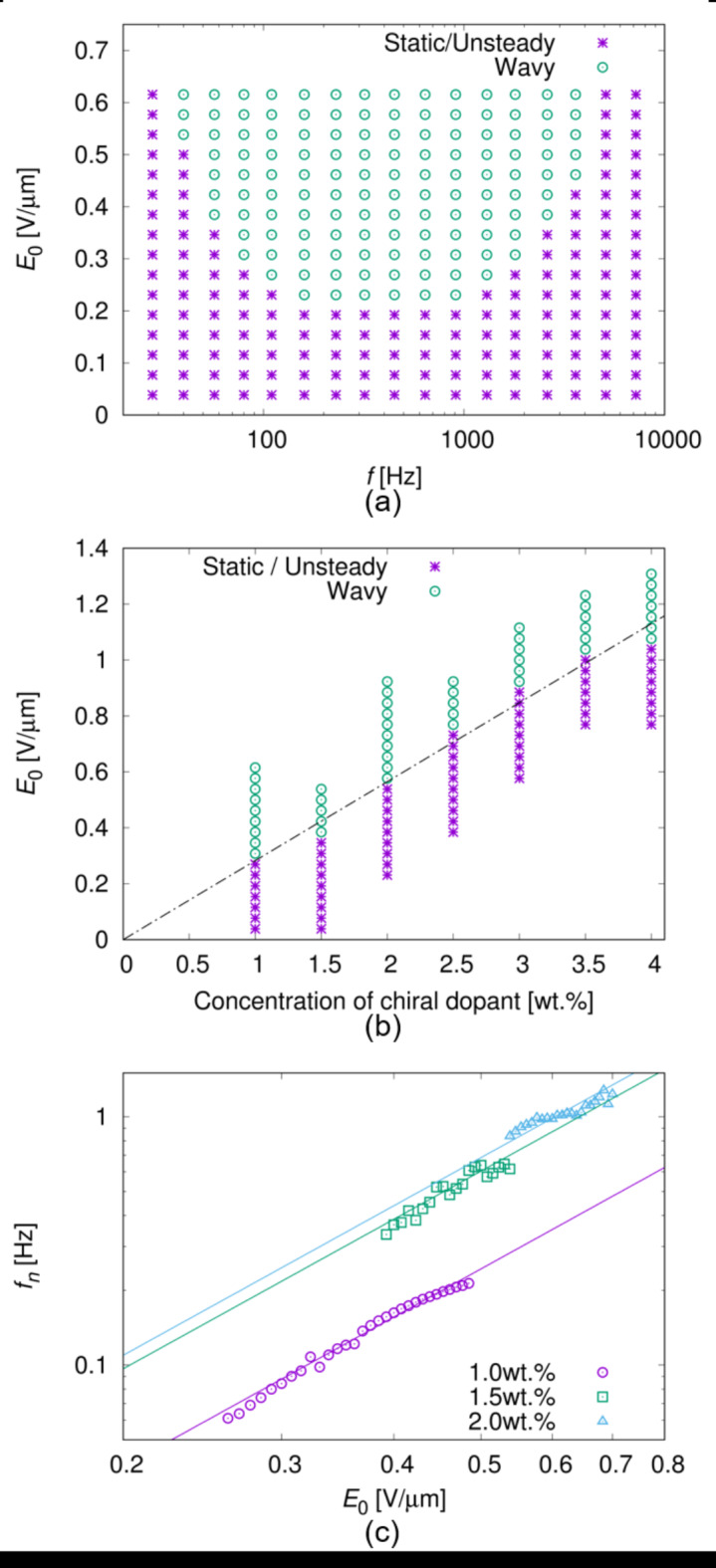
State diagrams and *E*_0_ dependence of *f*_*n*_. (**a**) shows the state diagram of the wave phenomenon with respect to the electric amplitude *E*_0_ and frequency *f*. Steady wave propagation was observed in a wavy state, and change in the texture is hardly observed or irregular motion was observed in a static/unsteady state (see Supplementary Video 2). The wavy state is realised when *E*_0_ is larger than a threshold amplitude *E*_*c*_ depending on *f*. In (**a**), the concentration of the chiral dopant was 1.0 wt% and the cell thickness was 13 μm. (**b**) shows the state diagram with respect to the electric amplitude *E*_0_ and the concentration of the chiral dopant. The threshold amplitude *E*_*c*_ proportionally increases with the increase of the concentration, as shown by the dashed line. (**c**) shows the *E*_0_ dependence of the wave frequency *f*_*n*_. The measurements were performed under different dopant concentrations of 1.0, 1.5, and 2.0 wt%. As shown by the solid lines, the data were fitted by quadratic functions of ${f_n} \propto E_{0}^{2}$. In (**b**) and (**c**), the cell thickness was 13 μm and *f* was 320 Hz.

We prepared a cylindrical droplet surrounded by a liquid solvent of PF656 in a sandwich cell with a homeotropic anchoring condition (see Fig. 1m and Methods section). The upper and lower surface of the droplet touch to the substrates, and the side surface touches to PF656. By applying an AC electric field of ${E_z}={E_0}\cos (2\pi ft)$ to the droplet, we observed the appearance of the fingerprint texture by polarising optical microscopy (POM), which has often been reported in Ch LC in sandwich cells^44–48^. We found that the fingerprint texture propagated in a plane parallel to the substrate, as shown in Fig. 1a–l and Supplementary Video 1. This indicates that the deformation of the director field propagates as a wave under an AC electric field. Wave propagation occurred unidirectionally or radially; in the latter case, a spiral pattern with three- or four-fold symmetry was formed, showing unidirectional rotation. The wave frequency *f*_*n*_ does not depend on the droplet diameter or whether the propagation is unidirectional or radial, as shown in Fig. 1n. The wave phenomenon with steady propagation, as shown in Fig. 1, is induced when the amplitude of the electric field *E*_0_ is larger than a threshold value as shown in the state diagrams of Fig. 2a,b. For the sake of convenience, we call the value as *E*_*c*_, and the “wavy state” as the situation where the steady wave propagation is induced. When *E*_0_ is smaller than *E*_*c*_, change in the texture was hardly observed or irregular motion was observed, and these situations are referred to as “static/unsteady state” (see also Supplementary Video 2). *E*_*c*_ depends on the AC frequency *f* and the concentration of the chiral dopant, as shown in Fig. 2a,b. The wavy state is realised when *f* is on the order of 10^1^–10^3^ Hz, and *E*_*c*_ proportionally increases with an increase in the concentration of the chiral dopant. By measuring the dependence of wave frequency *f*_*n*_ on electric amplitude *E*_0_ in the wavy state, we found that *f*_*n*_ is proportional to the square of *E*_0_, as shown in Fig. 2c.

**Fig. 3:**
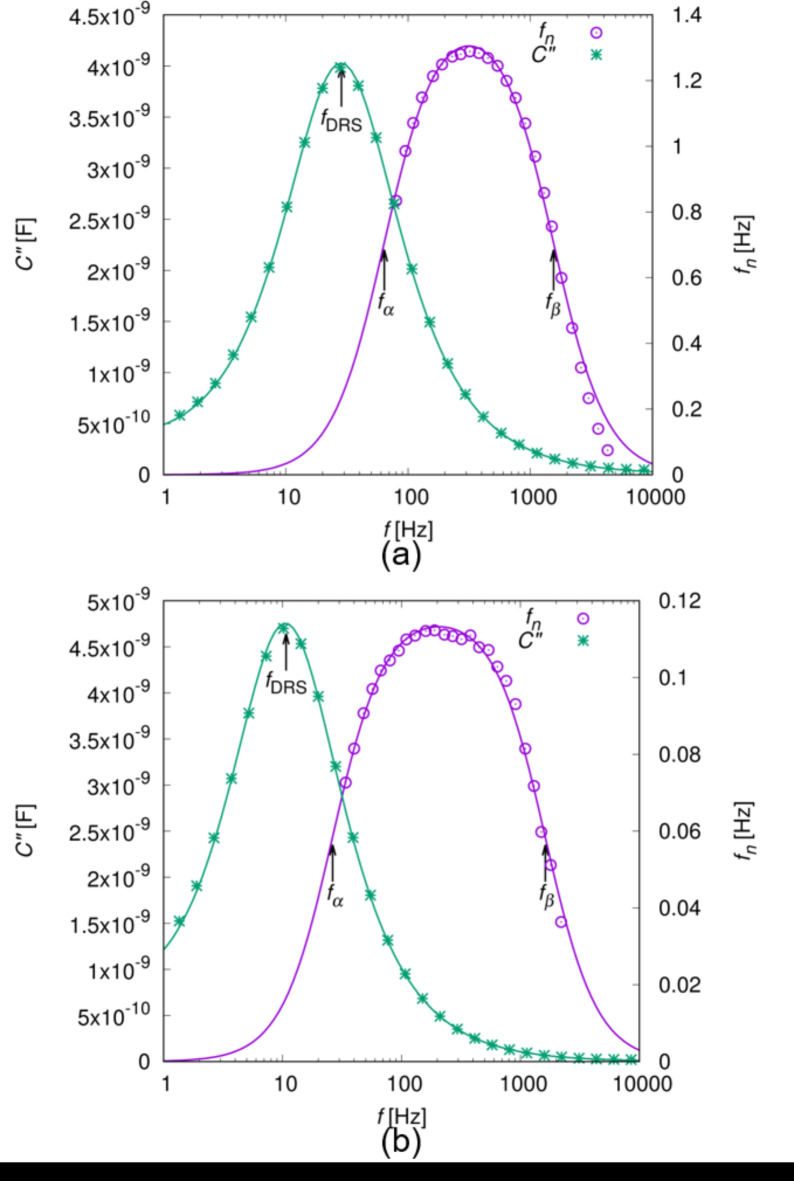
Dependence of *f*_*n*_ and *C*″ on the electric frequency *f*. The cell thickness *h* was 13 and 38 μm in (**a**) and (**b**), respectively. For the measurement of *f*_*n*_, the concentration of the chiral dopant was 2.0 and 0.5 wt% and the applied electric field was 0.85 and 0.24 V/µm in (**a**) and (**b**), respectively. *f*_*n*_ and *C*″ were fitted using Eqs. (19) and (23), respectively. The relaxations frequencies of *f*_DRS_, *f*_*α*_, and *f*_*β*_, are defined by ${1 \mathord{\left/ {\vphantom {1 {(2\pi {\tau _{{\text{DRS}}}})}}} \right. \kern-0pt} {(2\pi {\tau _{{\text{DRS}}}})}}$, ${1 \mathord{\left/ {\vphantom {1 {(2\pi {\tau _\alpha })}}} \right. \kern-0pt} {(2\pi {\tau _\alpha })}}$ and ${1 \mathord{\left/ {\vphantom {1 {(2\pi {\tau _\beta })}}} \right. \kern-0pt} {(2\pi {\tau _\beta })}}$, respectively. These frequencies were obtained by the fitting, and they are shown by the black arrows in (**a**) and (**b**).

**Fig. 4:**
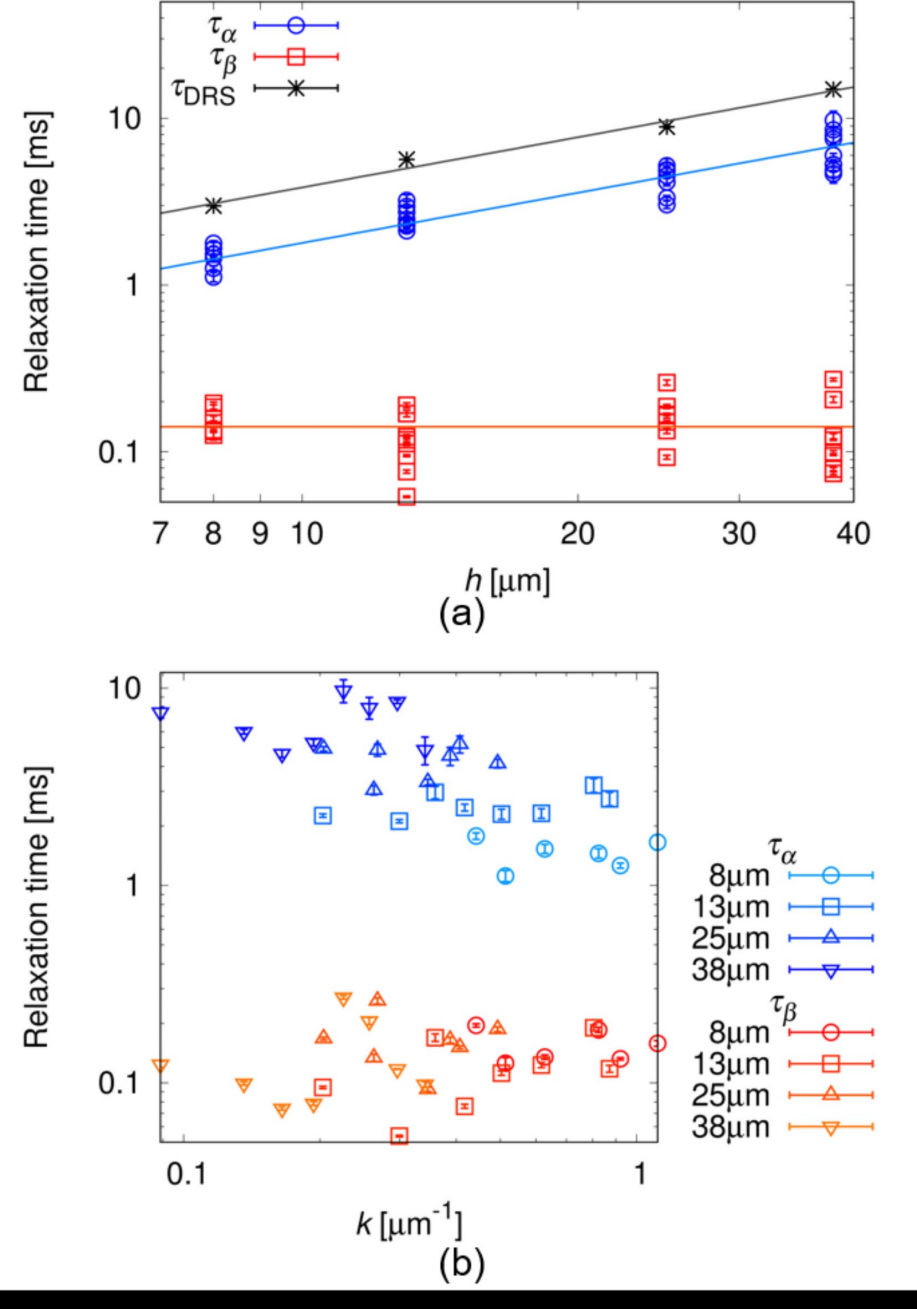
Dependence of relaxation times on *h* (**a**) and *k* (**b**). In both (**a**) and (**b**), measurements were performed in cells of *h* = 8, 13, 25 and 38 μm. *k* was measured in the fingerprint texture when *f* = 320 Hz. In (**a**), *τ*_*α*_ and *τ*_DRS_ were fitted by a linear function (${\tau _\alpha }={a_\alpha }h,\,\,{\tau _{{\text{DRS}}}}={a_{{\text{DRS}}}}h$) and *τ*_*β*_ was fitted by a constant (${\tau _\beta }=b$). The fitting parameters were obtained as ${a_\alpha }=0.179 \pm 0.008$ ms/µm, ${a_{{\text{DRS}}}}=0.385 \pm 0.012$ ms/µm and $b=0.141 \pm 0.010$ ms.

By measuring the dependence of the wave frequency *f*_*n*_ on the electric frequency *f*, we found that a broad peak appeared when *f* was on the order of 10^2^ Hz, as shown in Fig. 3a,b. In addition to this, the imaginary part of the electric capacity *C*″ (see the Methods section) was measured, to obtain the relaxation frequency of electrode polarisation process^41,42^. A sharp peak was observed in *C*″ in the order of 10^1^ Hz, which was one order lower than the peak frequency of *f*_*n*_. *C*″ showed a Debye-type relaxation, from which we obtained the relaxation time of *τ*_DRS_ (see Methods section). Because *τ*_DRS_ was proportional to the cell thickness *h* as shown in Fig. 4a, we can consider that the peak observed in *C*″ is attributed to the electrode polarisation process^41,42^. This suggests the presence of ionic impurities that migrated in the LC under an AC electric field.

When PF656 was not added into the sample, the wave phenomenon was not observed. Adding the PF656 into Ch LC, the phenomenon was induced; thus, it is considered that the ionic impurity in PF656 strongly contributes to the wave phenomenon. Adding small amount of PF656 into the Ch LC, and observing the cell filled with LC domain, we found that the wave phenomenon of the fingerprint texture was also induced under the AC electric field as shown in Supplementary Video 3. However, the fingerprint pattern was not observed in the whole region of the cell, and coexists with the dark region, where the director uniformly aligns parallel to the electric field (cell depth direction). In this situation, systematic control of the wave phenomenon was difficult, because the domain with the fingerprint texture moves (Supplementary Video 3), different from the case of the droplet (Fig. 1 and Supplementary Video 1). Therefore, we adopted the droplet geometry, to simplify the situation.

### Flow field measurement

**Fig. 5:**
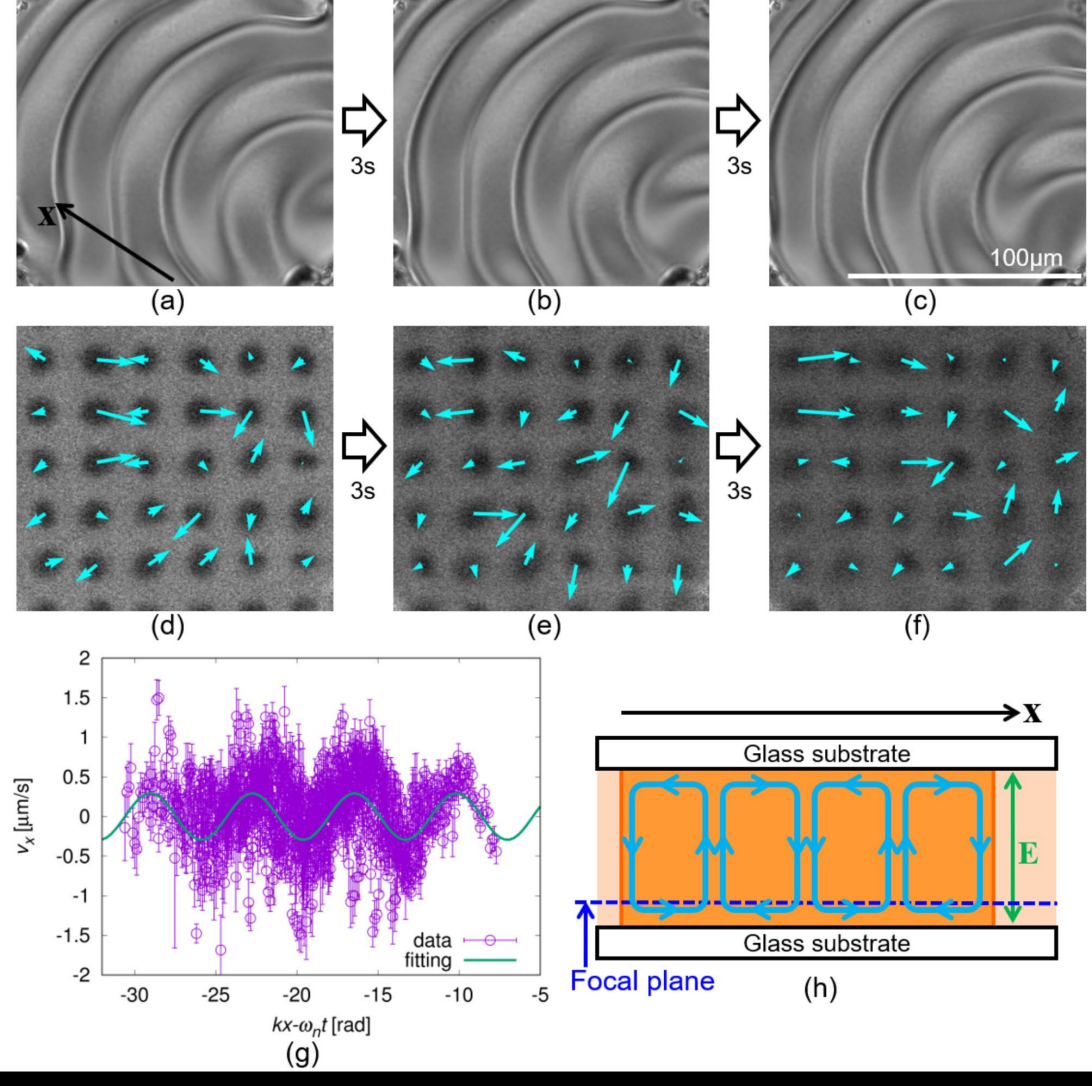
Measurement results of the flow field. (**a**–**c**) are POM images and (**d**–**f**) are confocal fluorescence images obtained after photo-bleaching, together with the flow field (aqua-coloured arrows) obtained by the measurement. The corresponding movie is available as Supplementary Video 4. The time interval in each image in (**a**–**c**) and (**d**–**f**) was 3 s. The black arrow in (**a**) indicates the direction of the wave propagation, and the white scale bar in (**c**) is 100 μm. The concentration of the chiral dopant was 0.5 wt% and the cell thickness *h* was 38 μm. In (**a**–**c**), the period of the fingerprint texture *λ* is ~ 50 μm, which is comparable with *h*. The dependence of the x-component of the flow velocity *v*_x_ on *kx* − *ω*_*n*_*t* is shown in (**g**). Here, *k* is the wavenumber of the fingerprint texture, *ω*_*n*_ is the angular frequency of the wave propagation, and *t* is time. The data was fitted using a sine function of ${v_x} \propto \sin (kx - {\omega _n}t+{\delta _0})$, where *δ*_0_ is the phase when *kx* − *ω*_*n*_*t =* 0. (**h**) shows a schematic of the flow field deduced from the measurements. The focal plane in the measurement was near the substrate, as shown by the blue dashed line. Periodic convection was considered to be induced as shown by the aqua-coloured lines, and the period of the convection is suggested to be *λ*.

The flow field in the wavy state was measured using the fluorescence photobleaching method^25,40^ (see Methods section), as shown in Fig. 5. Near the cell substrate in the cylindrical droplet (Fig. 5h), the flow field was inhomogeneous and unsteady, that is, the flow direction and speed depended on both space and time, as shown in Fig. 5d–f. Here, we define *k* as the wavenumber of the fingerprint texture and *ω*_*n*_ as the angular frequency of the wave propagation (*ω*_*n*_ = 2*πf*_*n*_); the x-axis is set as the direction of the wave propagation. Using the measurement results, we obtained the dependence of the x-component of the flow velocity *v*_*x*_ on *kx* − *ω*_*n*_*t*. As shown in Fig. 5g, *v*_*x*_ exhibits periodic behaviour fitted by a sine function (${v_x} \propto \sin (kx - {\omega _n}t+{\delta _0})$). This indicates that the flow field has a periodic structure and propagates in space, similar to the deformation of the director field. Considering the conservation of matter in a droplet, it is reasonable to assume the appearance of periodic convection (Fig. 5h), which has been observed during electroconvection in LC systems^2^. Periodic convection was considered to propagate together with the deformation of the director field.

## Discussion

### Simplification of flow, director, and concentration fields

The mechanism of the aforementioned wave phenomenon was analysed under the assumption that the phenomenon was due to electroconvection induced by the migration of ionic impurities in the LC. We consider that the electroconvection is induced by the same mechanism with the Carr-Helfrich instability, which has been well studied in the nematic (N) LC^2,4–20^. On the other hand, the director field twists spontaneously in the Ch LC used in this study, different from N LC. The helical structure due to the twist interacts with the velocity fields of LC and ionic impurities. We must discuss the director field of the LC (**n**), concentration (volume fraction) field of the ionic impurities (*ϕ*), and flow fields of both the LC and impurities (**v** and **c**). Exactly, these fields should be obtained by the solutions of the hydrodynamic equations, such as Ericksen-Leslie and advective-diffusion equations^2,35^. These equations are constructed in every place in the system, and the simultaneous equations obtained in this way should be solved. However, this is a very tough task because of the complexity of the equations. To avoid this complexity, we use the trial function method in this study. To simplify the situation, trial functions were designed for the flow, director, and concentration fields as discussed below, based on the experimental results and the Carr-Helfrich model.

For further simplification, in this study, we discuss only the case of unidirectional propagation along the x-direction. We assume that the propagation occurs uniformly, neglecting the contribution from the confinement effect in the cylindrical geometry. As shown in the Flow field measurement section, the presence of periodic convectional flow was suggested (Fig. 5). The flow field **v** can be described as follows^1,25,49^:

1 $$\begin{gathered} {v_x}={V_c}\sin \frac{{2\pi z}}{h}\sin ({k_m}x - {\delta _m}) \hfill \\ {v_y}=0 \hfill \\ {v_z}=\frac{{h{k_m}{V_c}}}{\pi }{\cos ^2}\frac{{\pi z}}{h}\cos ({k_m}x - {\delta _m}), \hfill \\ \end{gathered}$$

where the wavenumber of this periodic convection is *k*_*m*_, defined as,

2 $${k_m}=\frac{k}{m}.$$

**Fig. 6:**
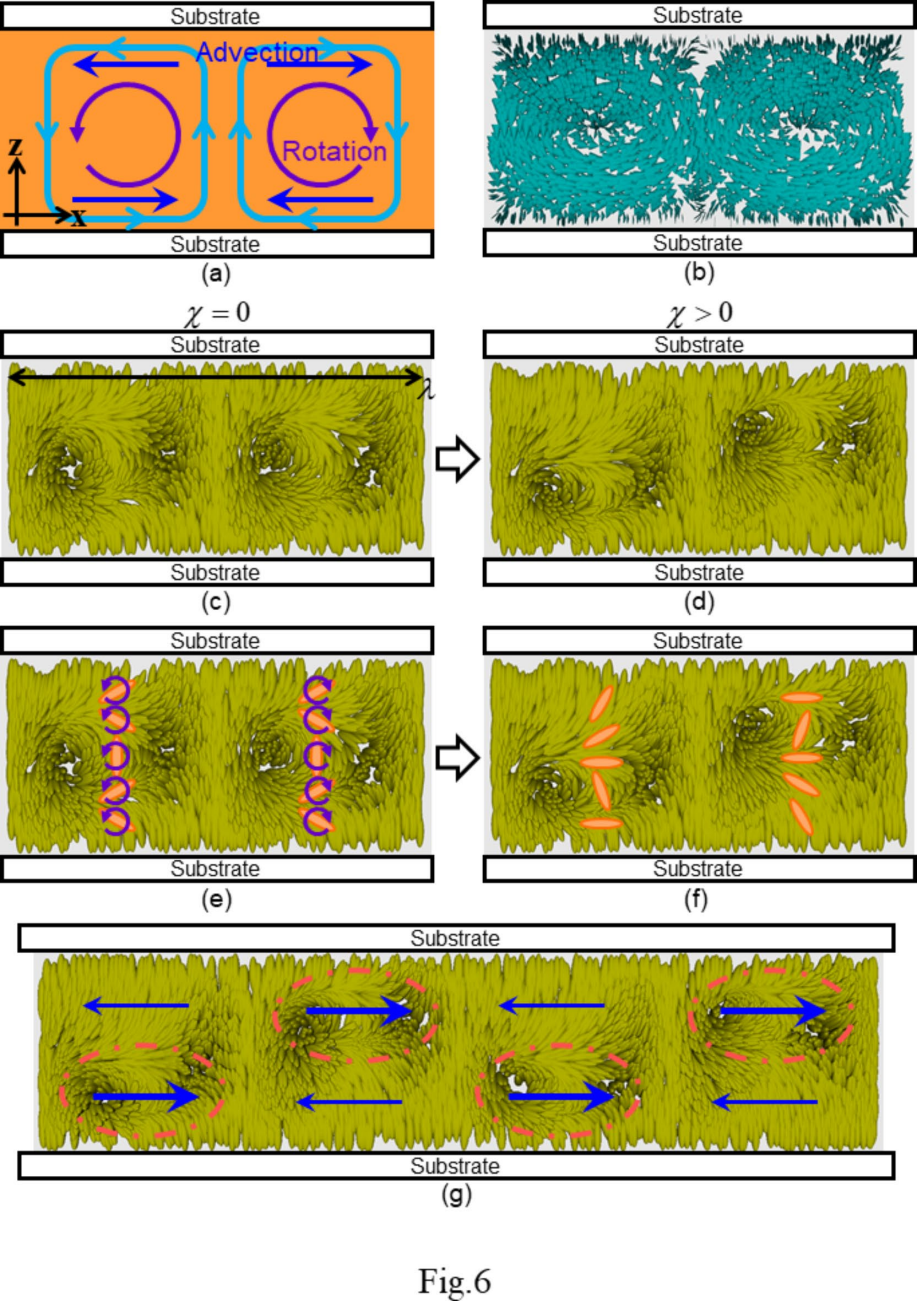
Schematics of the flow and director fields. The case of *m* = 1 in Eqs. (1)–(5) is shown. Circular flows shown by the aqua-coloured lines in (**a**) and cones in (**b**) were assumed in this study. (**b**) was obtained based on the trial function of Eq. (1). The director field based on Eqs. (3) and (4) are depicted in (**c**) and (**d**), where *χ* = 0 and *χ* > 0 are assumed, respectively. In (**c**), *λ* is defined as ${{2\pi } \mathord{\left/ {\vphantom {{2\pi } k}} \right. \kern-0pt} k}$, and the structural period of the director field is ${\lambda \mathord{\left/ {\vphantom {\lambda 2}} \right. \kern-0pt} 2}$. The deformation from (**c**) to (**d**) is induced by the flow. Owing to the circulating flows, the director rotates (purple arrows in (**a**) and (**e**)), resulting in the structural deformation as shown in (**f**). In this situation, large distortions of the director are periodically localised, as shown by the red ellipsoids in (**g**). Considering the advection along the x-axis, its direction changes periodically, as shown by the blue arrows in (**a**) and (**g**). However, in the region with a large distortion (red ellipsoids), where the advection strongly contributes to the director motion, the flow direction is always positive along the x-axis. Therefore, the entire director field propagates unidirectionally.

In this study, we assume that *m* is a positive integer for simplicity: the period of convection is an integral multiple of the structural period of the director field showing the fingerprint pattern. In Eq. (1), *h* is the cell thickness and the two cell substrates locate in the planes of *z* = ± *h*/2. Here, *δ*_*m*_ is assumed to be a time-dependent parameter (*δ*_*m*_(*t*)), and *V*_*c*_ is the characteristic flow velocity. Because the other parameters expect for *δ*_*m*_ and *V*_*c*_ are assumed to be time-independent, the periodic structure of the convection is always maintained. In the plane *z* = ± *h*/4, *v*_*x*_ changes periodically with the amplitude of *V*_c_. Equation (1) satisfies the incompressible (∇⋅**v** = 0) and non-slip conditions at the substrates (*v*_*x*_ = 0 for *z* = ± *h*/2)^1^. The influx and outflux are zero at the substrates (*v*_*z*_ = 0 for *z* = ± *h*/2). Schematics of the flow field described by Eq. (1) are presented in Fig. 6a,b.

The director field **n** when the fingerprint pattern appeared was analysed as described in Refs.^44–48^. Based on Ref.^44^, we designed the trial function of the director field in the present situation as follows:

3 $$\begin{gathered} {n_x}=\frac{{\sin 2\Theta }}{2}(1 - \cos 2(kx - \delta )) \hfill \\ {n_y}= - \sin \Theta \sin 2(kx - \delta ) \hfill \\ {n_z}= - {\cos ^2}\Theta - {\sin ^2}\Theta \cos 2(kx - \delta ), \hfill \\ \end{gathered}$$

where

4 $$\Theta =\frac{\pi }{2}\left( {1+\frac{z}{{2h}} - \chi \cos \frac{{\pi z}}{h}\sin ({k_m}x - {\delta _m})} \right).$$

In Ref.^44^, the director field is represented by the four parameters of *α*, *β*, *γ* and *ϕ*. Assuming that $\alpha =\beta =\pi$, $\gamma = - \Theta$ and $\phi =\pi +2(kx - \delta )$, we obtain Eqs. (3) and (4).

Equation (3) indicates the presence of a periodic structure with wavenumber 2*k*, as shown in Fig. 6c. We define *λ* as the structural period of the fingerprint texture observed by POM ($\lambda ={{2\pi } \mathord{\left/ {\vphantom {{2\pi } k}} \right. \kern-0pt} k}$, Fig. 1a) and assume that the structural period of the director field in Eq. (3) corresponds to ${\lambda \mathord{\left/ {\vphantom {\lambda 2}} \right. \kern-0pt} 2}$. Two dark lines are observed in one period of the texture (black arrows in Fig. 1a), and these are considered to correspond to the region with unidirectional alignment of the director along the z-axis. In Eq. (3), the alignment of (*n*_*x*_, *n*_*y*_, *n*_*z*_) = (0, 0, 1) is realised only when 2(*kx*−*δ*) is an integral multiple of 2*π*. This corresponds to the unidirectional alignment being realised twice in one period of the texture, consistent with the aforementioned observation results. To describe the texture more strictly, we need to add another deformation with wavenumber *k* into Eq. (3), while for simplicity we use Eq. (3) in this study.

In addition, we consider that the periodic deformation is induced by convective flow, as shown in Fig. 6d. The parameter *χ* in Eq. (4) indicates the degree of deformation, whose wavenumber is *k*_*m*_. To preserve the director field with this deformation during propagation, the following relationship should be satisfied between the time-dependent parameters *δ*_*m*_ and *δ*:

5 $${\delta _m}(t)=\frac{{\delta (t)}}{m}.$$

The director deformation mechanism is schematically illustrated in Fig. 6e,f. Deformation is induced by the rotational viscous force due to the existence of circulating flows (Fig. 6a,b)^2,24,49^. In this case, large distortions are periodically localised in the director field, as shown in Fig. 6g.

The flow also results in advection of the director^2,49^. In the flow field described by Eq. (1), the flow direction along the x-axis changes periodically, as shown in Fig. 6a,g. Because the torque induced by advection is proportional to the spatial variation of the director field, the advection of the director is strongly induced in regions where the director distortion is localised in the present situation. In these regions, because the flow direction is the same as shown in Fig. 6g, entire director field should propagate unidirectionally. Thus, we can consider that the propagation is induced by the coupling effect between the flow and director fields.

The concentration and flow fields of the ionic impurities (*ϕ* and **c**) should satisfy the continuum Eq^1^

6 $$\frac{{\partial \phi }}{{\partial t}}= - \nabla \cdot (\phi {\mathbf{c}}),$$

where *ϕ* is given by volume fraction.

**Fig. 7:**
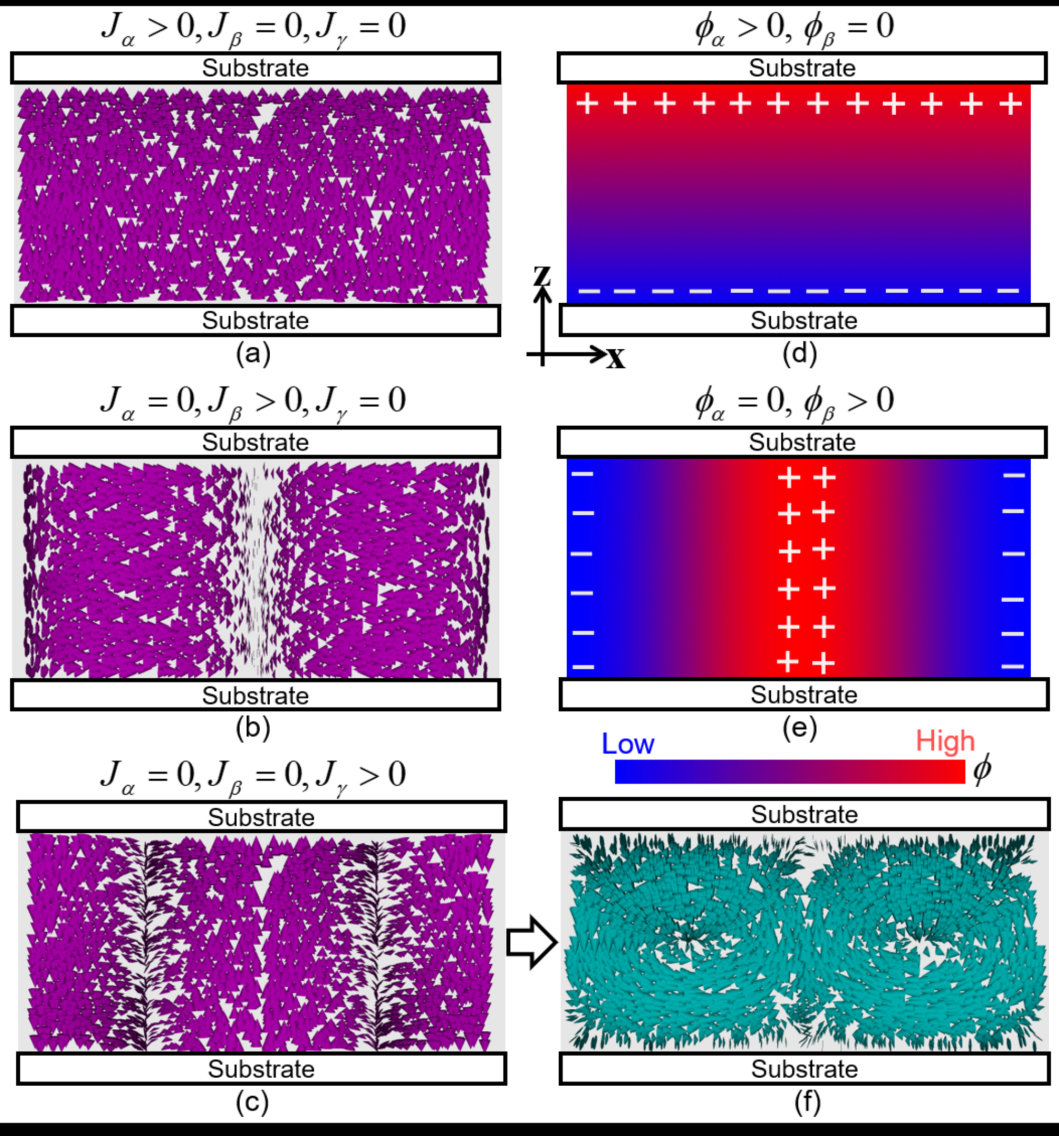
Schematics of the material flux and concentration fields of ionic impurities. The case of *m* = 1 in Eqs. (7) and (8) is shown. (**a**–**c**) indicate the flux fields based on the trial function of Eq. (8). The case of *J*_*α*_ > 0, *J*_*β*_ = 0, and *J*_*γ*_ = 0 is depicted in (**a**), *J*_*α*_ = 0, *J*_*β*_ > 0, *J*_*γ*_ = 0 in (**b**), and *J*_*α*_ = 0, *J*_*β*_ = 0, *J*_*γ*_ > 0 in (**c**); *J*_*δ*_ is set to zero in all cases. The flux of the impurities with a positive charge is assumed. (**d**) and (**e**) indicate the concentration fields based on the trial function of Eq. (7). The case of *ϕ*_*α*_ > 0, *ϕ*_*β*_ = 0 is depicted in (**d**) and *ϕ*_*α*_ = 0, *ϕ*_*β*_ > 0 in (**e**). *ϕ* is assumed to be the concentration of impurities with a positive charge. The high-concentration region is denoted in red, and the low-concentration region is indicated in blue. In the low *ϕ* region, the impurities with negative charges are considered to accumulate. Owing to the periodic flux of the impurities, the convection of LC is induced, as shown in (**c**) and (**f**).

According to the Carr-Helfrich model, when an electric field is applied along the z-direction, a concentration gradient of the ionic impurity is induced in this direction, owing to its migration to the electrode substrate (Fig. 7d). In addition, a periodic concentration gradient is induced in the direction perpendicular to the electric field in the LC system (Fig. 7e) owing to its anisotropic electrical conductivity^2^. This characteristic concentration field resulted in electroconvection. The trial functions of the concentration and flow fields should be given as simple forms, and satisfy the continuum equation of Eq. (6). Considering the discussion thus far, we designed trial functions for these fields as follows:

7 $$\phi ={\phi _0}+\frac{{2{\phi _\alpha }z}}{h}+{\phi _\beta }\cos ({k_m}x - {\delta _m}),$$

8 $$\begin{gathered} {c_x}= - \frac{1}{\phi }\left( {{J_\beta }+\frac{{2{J_\gamma }{\phi _\alpha }}}{{{\phi _0}}}} \right)\sin ({k_m}x - {\delta _m})+\frac{{{J_\delta }}}{\phi }\cos ({k_m}x - {\delta _m}) \hfill \\ {c_y}=0 \hfill \\ {c_z}=\frac{{{J_\alpha }}}{\phi }\left( {1 - \frac{{4{z^2}}}{{\xi h}}} \right)+\frac{{h{k_m}{J_\gamma }}}{{{\phi _0}}}\cos ({k_m}x - {\delta _m}). \hfill \\ \end{gathered}$$

In Eq. (7), the first term *ϕ*_0_ is a constant and the second term indicates the appearance of a concentration gradient along the z-axis, as shown in Fig. 7d. This term is proportional to *z*, showing *ϕ*_*α*_ and −*ϕ*_*α*_ at the cell substrate. The third term indicates the appearance of periodic modulation along the x-axis (Fig. 7e); its amplitude and wavenumber are *ϕ*_*β*_ and *k*_*m*_, respectively.

In Eq. (8), parameters *J*_*α*_, *J*_*β*_, *J*_*γ*_, and *J*_*δ*_ have the dimensions of the velocity, indicating the flux of the impurity. The terms *J*_*α*_ and *J*_*β*_ are designed to yield the concentration field of Eq. (7) under the constraint of Eq. (6) (Fig. 7a,b). From Eqs. (6)–(8), we obtain the following relationship between the concentration and flux:

9 $$\begin{gathered} {{\dot {\phi }}_\alpha }=\frac{4}{\xi }{J_\alpha } \hfill \\ {{\dot {\phi }}_\beta }={k_m}{J_\beta } \hfill \\ {\phi _\beta }{{\dot {\delta }}_m}={k_m}{J_\delta }, \hfill \\ \end{gathered}$$

where the dots indicate the time differentiation (${\dot {\phi }_\alpha }={{\partial {\phi _\alpha }} \mathord{\left/ {\vphantom {{\partial {\phi _\alpha }} {\partial t}}} \right. \kern-0pt} {\partial t}}$,${\dot {\phi }_\beta }={{\partial {\phi _\beta }} \mathord{\left/ {\vphantom {{\partial {\phi _\beta }} {\partial t}}} \right. \kern-0pt} {\partial t}}$, and ${\dot {\delta }_m}={{\partial {\delta _m}} \mathord{\left/ {\vphantom {{\partial {\delta _m}} {\partial t}}} \right. \kern-0pt} {\partial t}}$). Here, *J*_*δ*_ is introduced to satisfy Eq. (6) via Eq. (9). The terms that include *J*_*γ*_ in Eq. (8) indicate the appearance of a periodic flow, as shown in Fig. 7c. Because of this flux of ionic impurities, convection was induced in the LC (Fig. 7c,f).

**Fig. 8:**
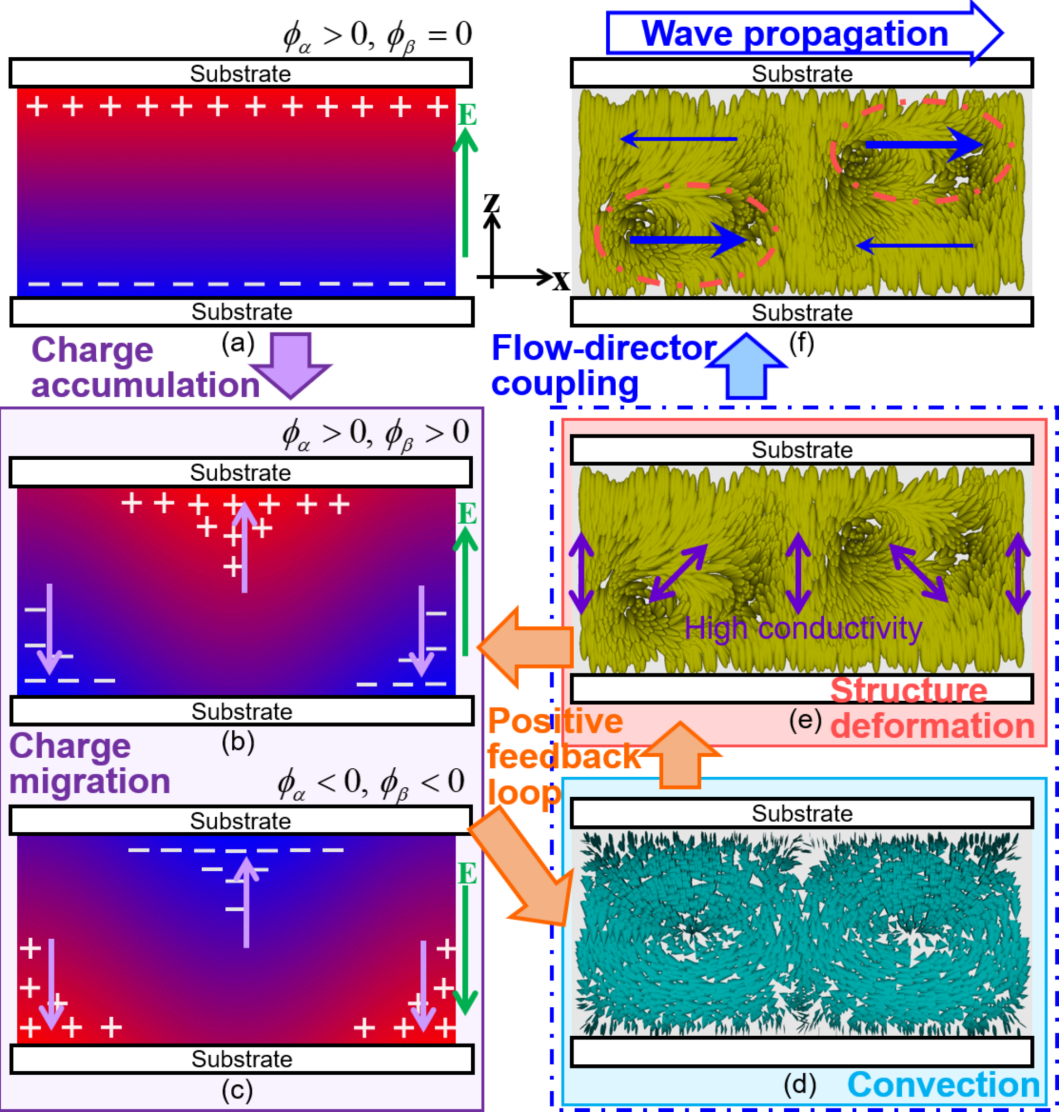
Schematics of the wave-generation mechanism. Upon applying an electric field, the ionic impurities migrate to the substrates, as shown in (**a**). In the director fields with the periodic deformation shown in (**e**), the direction with highest electric conductivity changes periodically in the x-direction, as indicated by the purple arrows in (**e**). In this situation, the impurities accumulate periodically along the x-axis, in addition to the migration to the substrates, as shown in (**b**) and (**c**). The accumulation under the electric field results in the periodic flux of the impurities, as illustrated by the light-purple arrows in (**b**) and (**c**). Here, because the distribution of the ionic impurity reverses when the electric field reverses, the direction of the periodic flux (light-purple arrows) is constant under the AC electric field. Owing to the flux of the impurity, the convection is induced in the LC, as shown in (**d**). The convection results in the director deformation of (**e**), and a positive feedback loop is realised between the charge migration, convection, and structure deformation. Transportation of the director field is induced by the coupling effect between the convection and the director deformation in the feedback loop, resulting in the wave generation shown in (**f**).

When an electric field is applied, *ϕ*_*α*_ appears because of the migration of ionic impurities to the electrode substrates (Fig. 8a). The appearance of *ϕ*_*β*_ results from the director deformation described above (Fig. 6d). Owing to this deformation and the anisotropy of the electrical conductivity of the LC, the direction with the highest conductivity changes periodically, as shown in Fig. 8e. This results in the periodic accumulation of impurities in addition to their migration to the substrates induced by the electric field (Fig. 8b). Because the accumulation is realised under an electric field, it results in the appearance of a material flux whose direction changes periodically, as shown in Fig. 8b (light-purple arrows). This flux is expressed by the appearance of *J*_*γ*_ in Eq. (8) (Fig. 7c). Because of the flux of impurities, convection was induced in the LC, as shown in Fig. 8d. Because we applied an AC electric field, the direction of the field depended on the time. When the electric field is reversed, the distribution of the ionic impurities is also reversed, as shown in Fig. 8b,c. Because both the field and distribution change, the sign of *J*_*γ*_, which indicates the direction of the periodic flux, does not change. Therefore, the direction of convection driven by this flux is constant under the AC field. Here, the distribution of the ionic impurity must periodically change, following the electric field as shown in (b) and (c). Strictly, this situation cannot be realised only by the effect of the electrostatic force, and the diffusion of the impurity should also be considered.

Summarising the discussion thus far, we suggest a model to describe the mechanism of wave generation under an electric field, as shown in Fig. 8. By applying an electric field to the Ch LC with the director deformation shown in Fig. 8e, the periodic accumulation of the ionic impurities dispersed in the sample is induced (Fig. 8b,c). This results in the periodic migration of impurities under an electric field, which induces convection of the LC, as shown in Fig. 8d. Owing to this convection, the director deforms, as shown in Fig. 8e; therefore, a positive feedback loop is formed between charge migration, convection, and structural deformation. The director field is transported by the coupling effect between the convection and director deformation in the feedback loop. Waves are produced owing to the unidirectional transportation of the periodic structure.

### Analysis based on the Onsager variational principle

The trial functions of Eqs. (1), (3), (7) and (8) do not satisfy the hydrodynamic equations of Ericksen-Leslie and advective-diffusion equations, because the functions are not the exact solutions of the equations. Instead of the use of these hydrodynamic equations, the validity of the model suggested above is examined by the application of the Onsager variational principle, which has been suggested as a unified theory to describe the dynamics of soft matter systems including LC^43,50,51^. According to this theory, the time evolution of the system is determined by minimising the Rayleighian ℜ, which is composed of the dissipation function *W* and the time differentiation of free energy $\dot {F}$, that is, $\Re =W+\dot {F}$. In the present situation, the Rayleighian about both ionic impurities and LC should be considered, and we call them ℜ_*c*_ and ℜ_*v*_, respectively.

For ℜ_*c*_, we consider three contributions: dissipation due to the friction between the impurities and LC, and the free energies of the mixing entropy and electrostatic potential. We denote the density of them as *w*_c_, *f*_*m*_ and *f*_*e*_, respectively, and they are described below based on the discussion in Refs.^51–54^. :

10 $${w_c}=\frac{{{\zeta _0}}}{2}\phi {({c_i} - {v_i})^2} - \frac{{\Delta \bar {\zeta }}}{2}\phi {\left( {{n_i}({c_i} - {v_i})} \right)^2},$$

11 $${f_m}=\frac{{{k_B}T}}{{{v_b}}}\phi \ln \phi,$$

12 $${f_e}= - {\rho _e}{E_i}{r_i}\phi.$$

Here, the suffix *i* denotes *x*, *y* and *z*, and each component of the positional vector is denoted as *r*_*i*_. In Eq. (10), *ζ*_0_ and $\Delta \bar {\zeta }$ are the effective friction constants between the impurity and LC^54^. The first term is the isotropic part, and the second term is the anisotropic part owing to the anisotropy of the LC. In a typical calamitic LC, $\Delta \bar {\zeta }$ should show a positive value, indicating that the friction acting on the impurity is the lowest when it migrates along the director. The anisotropy of electrical conductivity is attributed to this effect in this model. *k*_*B*_, *T*, and *v*_*b*_ in Eq. (11) are the Boltzmann constant, temperature, and volume of one impurity molecule, respectively, and *ρ*_*e*_ in Eq. (12) is the charge density. Equation (11) can be attributed to the mixing entropy in a dilute solution, and this is required to incorporate the diffusion effect of the impurity. In Eq. (12), the AC electric field **E** should be applied uniformly along the z-axis in the present situation.

13 $$({E_x},{E_y},{E_z})=(0,0,{E_0}\cos \omega t),$$

where *ω* = 2*πf*.

Considering the translational symmetry, Eqs. (10)–(12) should be integrated in the characteristic box region of *L*_*x*_*L*_*y*_*L*_*z*_, where *L*_*x*_ is chosen as 2*π*/*k*_*m*_, which is the period of convection, and *L*_z_ equals *h* of the cell thickness. After integration, ℜ_*c*_ is normalised by *L*_*x*_*L*_*y*_*L*_*z*_. Since *ϕ*, *c*, **v** and **n** are independent of *y*, we obtain:

14 $${\Re _c}=\frac{{{k_m}}}{{2\pi h}}\iint {\left( {\frac{{{\zeta _0}}}{2}\phi {{({c_i} - {v_i})}^2} - \frac{{\Delta \bar {\zeta }}}{2}\phi {{\left( {{n_i}({c_i} - {v_i})} \right)}^2}+\frac{{{k_B}T}}{{{v_b}}}({c_i} - {v_i})\frac{{\partial \phi }}{{\partial {r_i}}} - {\rho _e}{E_i}{c_i}\phi } \right){\text{dxdz}}},$$

where we used Eq. (6) and the incompressible condition of ∇·**v** = 0 (for more detail, see Supplementary Note or Refs.^51–54^).

For ℜ_*v*_, only the contribution of viscous dissipation *w* was considered in this study. According to Refs.^43,50^, it is described as

15 $${\Re _v}=\frac{{{k_m}}}{{2\pi h}}\iint {w{\text{dxdz}}},$$

16 $$w=\frac{1}{2}{\beta _1}{({e_{ij}}{n_i}{n_j})^2}+\frac{1}{2}{\beta _2}e_{{ij}}^{2}+\frac{1}{2}{\beta _3}{\left( {{e_{ij}}{n_j}} \right)^2}+\frac{1}{2}{\gamma _1}N_{i}^{2} - {\bar {\gamma }_2}{N_i}{e_{ij}}{n_j},$$

17 $${e_{ij}}=\frac{1}{2}\left( {\frac{{\partial {v_i}}}{{\partial {r_j}}}+\frac{{\partial {v_j}}}{{\partial {r_i}}}} \right),\,\,\,{N_i}=\frac{{\partial {n_i}}}{{\partial t}}+{v_j}\frac{{\partial {n_i}}}{{\partial {r_j}}} - \frac{1}{2}\left( {\frac{{\partial {v_i}}}{{\partial {r_j}}} - \frac{{\partial {v_j}}}{{\partial {r_i}}}} \right){n_j},$$

where *i* and *j* denote *x*, *y*, or *z*. *β*_1_, *β*_2_, *β*_3_, *γ*_1_, and *γ*_2_ are the viscosity coefficients and ${\bar {\gamma }_2}$ is defined as −*γ*_2_, which has a positive value in a typical calamitic LC^2^.

Under the assumptions of the flow, director, and concentration fields in the previous section, the total Rayleighian (ℜ = ℜ_*c*_ + ℜ_*v*_) is calculated. Using Eqs. (1)–(5), (7), (8), (13)–(17), and expanding them to the second-order of *ϕ*_*α*_, *ϕ*_*β*_, *J*_*α*_, *J*_*β*_, *J*_*γ*_, *J*_*δ*_, *V*_*c*_, we obtain

18 $$\begin{aligned} \Re &\sim {Z_\alpha }J_{\alpha }^{2}+{Z_\beta }J_{\beta }^{2} - {Z_{\alpha \beta }}\chi {J_\alpha }{J_\beta }+{h^2}k_{m}^{2}{Z_\gamma }J_{\gamma }^{2} - {h^2}k_{m}^{2}{Z_{\gamma c}}{\phi _0}{J_\gamma }{V_c} \hfill \\ &\quad +\frac{1}{h}{M_\alpha }{J_\alpha }{\phi _\alpha }+{k_m}{M_\beta }{J_\beta }{\phi _\beta } - {\rho _e}{E_0}\left( {{J_\alpha }+\frac{{h{k_m}}}{{2{\phi _0}}}{J_\gamma }{\phi _\beta }} \right)\cos \omega t \hfill \\ &\quad +\frac{1}{{{h^2}}}\eta V_{c}^{2} - \frac{1}{h}{\gamma _\chi }{V_c}\dot {\chi }+\frac{{13{\pi ^2}}}{{256}}{\gamma _1}{{\dot {\chi }}^2} - k{\gamma _\omega }\chi {V_c}\dot {\delta }+{\gamma _1}{{\dot {\delta }}^2}, \hfill \\ \end{aligned}$$

where *Z*_*α*_, *Z*_*β*_, *Z*_*αβ*_, *Z*_*γ*_, *Z*_*γc*_, *M*_*α*_, *M*_*β*_, and *γ*_*ω*_ are constants, and *η* and* γ*_*χ*_ are parameters dependent on *hk* and *hk*_*m*_ (for more details, see Supplementary Note).

By minimising Eq. (18) using *J*_*α*_, *J*_*β*_, *J*_*γ*_, *V*_*c*_, $\dot {\chi }$, and $\dot {\delta }$, we obtain six equations for these parameters ((S11), (S13a), (S13b), (S17a), (S17b) and (S19) in Supplementary Note). These equations indicate the diffusion effect of ionic impurities under electrostatic potential and the balance of the hydrodynamic forces acting to LC in the present model. Under the constraint of Eq. (9), these equations are solved (for more details, see Supplementary Note). Assuming that *χ* is time-independent and that the wave frequency of *f*_*n*_ is obtained by the time average of $\dot {\delta }$, we finally obtain

19 $${f_n}=\frac{1}{{2\pi }}\mathop {\lim }\limits_{{T \to \infty }} \frac{1}{T}\int_{0}^{T} {\dot {\delta }{\text{dt}}} \sim {f_{n0}}\frac{{{\tau _\alpha }({\tau _\alpha }+{\tau _\beta }){\omega ^2}}}{{(1+\tau _{\alpha }^{2}{\omega ^2})(1+\tau _{\beta }^{2}{\omega ^2})}},$$

where

20a $${\tau _\alpha }=\frac{{{Z_\alpha }\xi h}}{{2{M_\alpha }}},$$

20b $${\tau _\beta }=\frac{{2{Z_\beta }}}{{{M_\beta }k_{m}^{2}}},$$

21 $${f_{n0}}=\frac{{\rho _{e}^{2}E_{0}^{2}{Z_{\alpha \beta }}{Z_{\gamma c}}{\gamma _\omega }{\chi ^2}}}{{128\pi {Z_\alpha }{M_\beta }{Z_\gamma }{\gamma _1}}}\frac{{{h^3}k}}{\eta }.$$

Equation (19) is similar to the Debye-type relaxation observed in *C*″ in the DRS, whereas it is characterised by two relaxation times of * τ*_*α*_ and *τ*_*β*_, different from the Debye-type. *τ*_*α*_ and *τ*_*β*_ indicate the migration speeds of the impurities along z- and x-axes, respectively.

Equations (19) and (21) indicate that *f*_*n*_ is proportional to the square of the electric field amplitude (${f_n} \propto E_{0}^{2}$), which agrees with the experimental results shown in Fig. 2c. Moreover, the measurement results of the dependence of *f*_*n*_ on the electric frequency *f* are well fitted by Eq. (19), as shown in Fig. 3, and *τ*_*α*_, *τ*_*β*_, and *f*_*n*0_ are obtained from the fitting. As shown in Fig. 4a, *τ*_*α*_ is proportional to the cell thickness *h*, as well as the relaxation time of *τ*_DRS_ in the electrode polarisation process. Because both *τ*_*α*_ and *τ*_DRS_ are on the order of 10^0^ ms, it is reasonable to consider that *τ*_*α*_ is attributed to the migration of the ionic impurity along the z-direction. No dependence on *k* was observed in *τ*_*α*_, as shown in Fig. 4b.

**Fig. 9:**
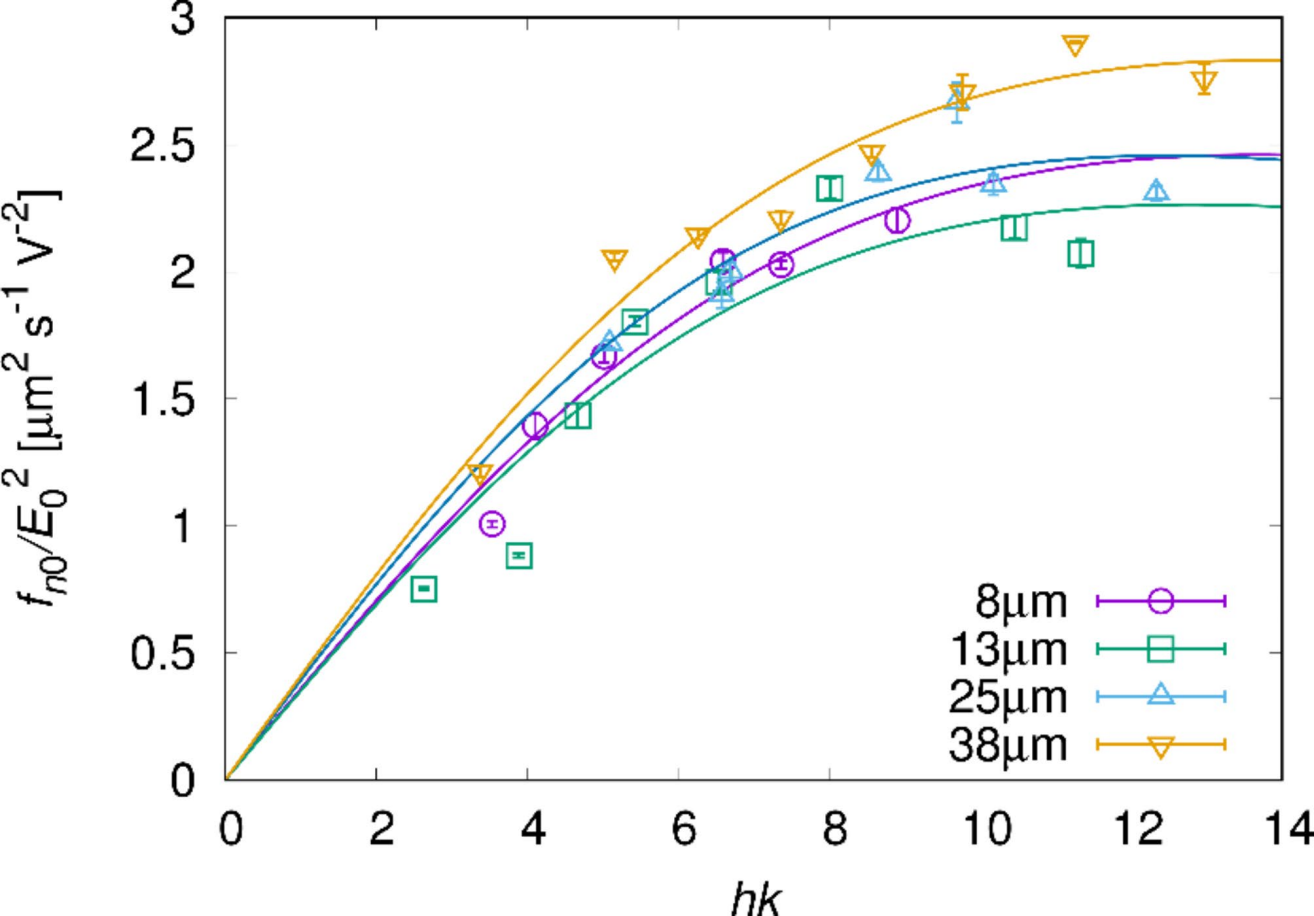
Dependence of the normalised wave frequency on parameter *hk*. The measurement was performed in cells with *h* = 8, 13, 25, and 38 μm. *k* was measured in the fingerprint texture when *f* = 320 Hz. The data were fitted by Eq. (22) for each *h* value. The fitting parameters were obtained as $$X=0.360 \pm 0.027\,\upmu{{\text{m}}^{\text{2}}}{{\text{s}}^{{\text{-1}}}}{{\text{V}}^{{\text{-2}}}}$$, $$Y=0.0054 \pm 0.0019$$ for *h* = 8 μm, $$X=0.353 \pm 0.039\,\upmu{{\text{m}}^{\text{2}}}{{\text{s}}^{{\text{-1}}}}{{\text{V}}^{{\text{-2}}}}$$, $$Y=0.0061 \pm 0.0022\,$$ for *h* = 13 μm, $$X=0.395 \pm 0.035$$$$\upmu{{\text{m}}^{\text{2}}}{{\text{s}}^{{\text{-1}}}}{{\text{V}}^{{\text{-2}}}}$$, $$Y=0.0065 \pm 0.0016\,$$ for *h* = 25 μm and $$X=0.411 \pm 0.021$$$$\upmu{{\text{m}}^{\text{2}}}{{\text{s}}^{{\text{-1}}}}{{\text{V}}^{{\text{-2}}}}$$, $$Y=0.0053 \pm 0.0008\,$$ for *h* = 38 μm.

*τ*_*β*_ does not depend on either *h* or *k*, as shown in Fig. 4a,b. Based on Eq. (20b), we consider *k*_*m*_ to be constant in the present situation. Because the parameters * χ* and *η* in Eq. (21) depend on *hk* and *hk*_*m*_, *f*_*n*0_ should be a function of *hk* and *h* under a constant *k*_*m*_. By normalising Eq. (21) using $E_{0}^{2}$, we obtain

22 $$\frac{{{f_{n0}}}}{{E_{0}^{2}}}=\frac{{Xhk}}{{1+Y{h^2}{k^2}}},$$

where *X* and *Y* are *h*-dependent parameters (for further details, see Supplementary Note). The measurement results of *hk* dependence of *f*_*n*0_ are well fitted by Eq. (22), as shown in Fig. 9. Because *k*_*m*_ does not depend on *k*, *m* should be proportional to *k* ($m \propto k$). However, the determination mechanism of *k*_*m*_ and *m* is not obtained by our model, so that the mechanism remains to be solved. To obtain it, we should consider the effects oversimplified or neglected in the model.

In the above analysis, the appearance of the wave phenomenon is described by four terms in the Rayleighian: the electrostatic potential, mixing entropy, friction between the ionic impurity and LC, and viscous dissipation of LC. It should be noted that our model does not consider the dielectric anisotropy and elasticity of LC. This indicates that these effects are not necessarily required to describe the mechanism of the present wave propagation phenomenon in the director field, although both the dielectric and elastic properties can contribute to the deformation of the director. Neglect of these effects significantly helped to simplify our model and to provide a possible description of wave propagation. However, a discussion that includes these effects would be necessary to clarify the details of the present phenomenon, such as the determination of the threshold electric field for wave generation and the propagation configuration.

We assumed the flow, director and concentration fields by using the trial functions of Eqs. (1), (3), (7) and (8), and did not discuss why these formulae were derived theoretically. It is significant to describe the present wave phenomenon without the above assumptions, starting from the hydrodynamic equations or minimisation of the Rayleighian. To perform this, much more complicated analysis would be necessary, compared to the simplified analysis in this study. Because exact analytical solutions might not be obtained, numerical analysis should also be performed.

## Conclusion

In this study, we observed a wave phenomenon in which the director field of a Ch LC propagated under an AC electric field. Assuming that this phenomenon was induced by electroconvection, we also analysed the LC flow and migration of the ionic impurities of the charge carrier. The flow-field and DRS measurements revealed the existence of the convective flow propagating together with the director and the ionic impurities migrating under the electric field, respectively; these measurement results indicated the presence of the electroconvection. Based on the experimental results, we proposed a possible model for describing the wave generation mechanism, as illustrated in Fig. 8. In this model, convection is stabilised by the interaction between the concentration, flow, and director fields via the formation of a positive feedback loop. Finally, the wave is produced by the interaction between the convective flow and the deformed director stabilised in the feedback loop.

By simplifying the concentration, flow, and director fields using trial functions, we examined the validity of the above model based on Onsager variational principle. Consequently, the dependence of the wave frequency *f*_*n*_ on the electric frequency *f* exhibits a broad peak shape function, which agrees with the measurement results of *f*_*n*_. In addition, our model suggests that *f*_*n*_ is proportional to the square of the electric field (${f_n} \propto E_{0}^{2}$), which agrees with the experimental results. Because the model accurately describes the properties of the wave propagation phenomenon, it should be considered that this phenomenon is induced by electroconvection.

In the analysis, we considered four effects: the electrostatic potential, mixing entropy, friction between the ionic impurity and LC, and viscous dissipation of the LC. It should be noted that only the third and fourth effects in this list result from the characteristic properties of the LC. These effects, which indicate anisotropic dissipations in the LC system, mainly contribute to the stabilisation of convection and wave generation. Although the mechanism of the electroconvection of LC is complicated, it can mainly be attributed to the minimisation of the anisotropic energy dissipations.

## Methods

### Sample preparation

As the host N LC, we used E8 (Merck Ltd.) with positive dielectric and conductivity anisotropy. The chiral dopant (S)-2-Octyl 4-[4-(hexyloxy)benzoyloxy]benzoate (TCI) was added to E8 at a concentration of 0.5–4.0 wt% to make Ch LC. To prepare the droplets, we used the fluorinated oligomer PF656 as an isotropic liquid solvent (OMNOVA Solutions Inc.). The Ch LC sample was added to PF656 at concentrations of 25–30 wt%, and we made the droplets. These materials were mixed with each other at high temperatures (~ 100 °C) and exhibited macroscopic phase separation at room temperature^55^. By cooling the sample from a well-mixed state, Ch LC droplets dispersed in an isotropic solvent were obtained. For fluorescence microscopy in the flow field measurements, the fluorescent dye poly[tris(2,5-bis(hexyloxy)-1,4-phenylenevinylene)-alt-(1,3-phenylenevinylene)] (Sigma-Aldrich Co., LLC) was added to the sample at a concentration of 0.01 wt%. For DRS, we prepared an N LC by mixing E8 and PF656 in a weight ratio of 97:3.

### Application of electric field and polarised microscopy

To apply an electric field to the Ch LC droplets, sandwich cells were prepared using two ITO-coated glass substrates and film spacers. The substrates were coated with a fluorinated resin, CYTOP (Asahi Glass Co., Ltd.), which facilitated strong homeotropic anchoring^56^. As the spacers, we used polyimide or PET films, whose thickness was 8–38 μm. In this study, because we focused on droplets whose diameters 2*R* were sufficiently larger than the cell thickness (2*R* ~ 50–250 μm), it should be considered that they had a pillar shape rather than a spherical shape as shown in Fig. 1 m. After applying an alternating-current (AC) electric field to the samples, we observed them using polarised microscopy (BH2, Olympus Co., or ECLIPSE LV100, Nikon Co.). The electric field is applied along the direction normal to the cell substrates, which is defined as the z-direction in this study. Thus, the field is described as Eq. (13).

### Dielectric relaxation spectroscopy

For the DRS measurements, we used the N LC sample and the sandwich cells described above. The complex electric capacitance C^*^ of the cell filled with the sample was measured using a commercial impedance analyser (Alpha-A high-performance frequency analyzer, Novocontrol Technologies GmbH & Co. KG). Considering that C^*^ is affected by direct-current (DC) conduction and electrode polarisation due to the migration of ionic impurities in the sample, the imaginary part of the capacitance *C*″ can be described as^41,42^,

23 $$C^{\prime\prime}={C_{{\text{DC}}}}{\omega ^{ - {m_{{\text{DC}}}}}}+\frac{{{C_{{\text{EP}}}}\omega {\tau _{{\text{DRS}}}}}}{{1+{{(\omega {\tau _{{\text{DRS}}}})}^2}}},$$

where *τ*_DRS_ is the relaxation time of the polarisation and *C*_DC_, *C*_EP_, and *m*_DC_ are constants. By fitting the measurement results using Eq. (23), we successfully obtained *τ*_DRS_, as shown in Fig. 3.

### Flow-field measurement by the fluorescence photobleaching method

The flow field was measured using the previously described fluorescence photobleaching method^25,40^. A fluorescent LED illumination system (D-LEDI, Nikon Co., Ltd.) was used as the light source for photobleaching. First, the sample was bleached into a lattice pattern using strong light illumination through a photomask. The flow distribution was obtained from the time evolution of fluorescence images after photobleaching. Images were obtained using a confocal microscopy system constructed with a MAICO MEMS confocal unit (Hamamatsu Photonics Co., Ltd.) and an inverted microscope (Ti2-U, Nikon Co., Ltd.). In this study, we measured the flow field near the cell substrate, setting the distance between the substrate and focal plane to ~ 5 μm in a 38-µm-thick cell.

### Analytical calculation

Analytical calculations were performed using the commercial software Mathematica (Wolfram Research, Inc.), and three-dimensional drawings of the director and flow fields based on the trial functions were created using the freely available POV-Ray software.

## Electronic supplementary material

Below is the link to the electronic supplementary material.

Supplementary Material 1

Supplementary Material 2

Supplementary Material 3

Supplementary Material 4

Supplementary Material 5

## Data Availability

All data supporting the findings of this study are available in the article and Supplementary Information. Additional information is available from the corresponding author on request.
