# Supplementary material for: Propagation of periodic director and flow patterns in a cholesteric liquid crystal under electroconvection
Source: Sci Rep. 2024 Oct 5;14:23201. doi: 10.1038/s41598-024-74551-w (PMC11457521; doi:10.1038/s41598-024-74551-w)
Supplement: Supplementary file 5 — Supplementary Material 5 [file 41598_2024_74551_MOESM5_ESM.docx]

**Propagation of periodic director and flow patterns in a cholesteric liquid crystal under electroconvection**

Jun Yoshioka, Hiroki Nobori, Koji Fukao and Fumito Araoka

**Supplementary Note:**

1. Legends for videos
2. Derivation of equation (14)
3. Derivation of equation (19)

**1. Legends for videos**

**Video 1.** Real-time movie of propagation of fingerprint texture under an AC electric field in cylindrical Ch LC droplets. Experimental condition is the same with Fig.1 (a)–(l), and they are described in the legend of Fig.1. Unidirectional propagation was observed in (a) and radial propagation in (b) and (c). The spiral structures with three- and four-fold symmetry are formed in (b) and (c), respectively, together with a steady rotation.

**Video 2.** Real-time movie of static/unsteady and wavy states under POM observation. (a), (b) and (c) are static, unsteady and wavy states, respectively. Concentration of the chiral dopant is 2.0wt.%, and cell thickness is 13 μm. Electric amplitude *E*_0_ is 0.69 V/ μm; the frequency *f* is 10 Hz, 110Hz and 320Hz in (a), (b) and (c), respectively.

**Video 3.** Real-time movie of propagation of fingerprint texture under an AC electric field in a cell filled with Ch LC. The sample was observed by POM. The concentration of the chiral dopant was 1.0 wt.% in Ch LC and the weight ratio of Ch LC and PF656 was 9:1. Cell thickness was 20 μm, and the amplitude and frequency of the applied electric field were 0.50 V/μm and 320 Hz, respectively (*E*_0_ = 0.34 V and *f* = 320 Hz). A sine wave was applied as the field.

**Video 4.** Real-time movie of flow field measurement with photo-bleaching method. Experimental condition is the same with Fig.5 (a)–(f), and they are described in the legend of Fig.5. (a) is obtained by POM observation, and (b) is by confocal fluorescence microscopy after photo-bleaching. The time evolution of flow field is depicted in (c) together with the fluorescence image.

**2. Derivation of equation (14)**

To obtain ℜ*_c_*, equations (10)–(12) in the main text should be integrated in a characteristic box region of $L_{x}L_{y}L_{z}$, where *L_x_* should be chosen as 2*π*/*k_m_*, which is the period of the convection, *L*_z_ should be *h* of cell thickness. After the integration, ℜ*_c_* is normalised by $L_{x}L_{y}L_{z}$:

, (S1)

where we assumed that the system has continuous translational symmetry along y-axis.

The time differentiation of and is given by total differentiation:

, (S2a)

, (S2b)

where suffix *i* indicates *x*, *y* or *z*; the differentiation by these coordinates is indicated by .

Using equations (6), (11) and (S2a), we obtain the integral of  as,

, (S3)

where we used the partial integral and neglected the surface integral term. After that, the incompressible condition of $\partial_{i}v_{i}=0$ was used.

In the similar way, the integral of is obtained by the use of equations (6), (12) and (S2b);

. (S4)

Using equations (10), (S1), (S3) and (S4), we obtain equation (14).

**3. Derivation of equation (19)**

Using equations (1)–(5), (7), (8), (13)–(17) in the main text, we calculated the Rayleighian ℜ. Expanding it to the first order of *χ*, we obtain

, (S5)

where we neglected the terms higher than the second order of *φ_α_*, *φ_β_*, *J_α_*, *J_β_*, *J_γ_* and *V_c_*, and the terms higher than or equal to the first order of *h*/*ξ*. The parameters *Z_α_*, *Z_β_*, *Z_αβ_*, *Z_γ_*, *Z_γc_*, *M_α_*, *M_β_*, are given by,

, (S6a)

, (S6b)

, (S6c)

, (S6d)

. (S6e)

, (S7a)

. (S7a)

The parameter *η* depends on *hk* and *hk_m_*:

$\eta=A+Bh^{2}k^{2}+Ch^{2}k_{m}^{2}+Dh^{4}k_{m}^{4}$, (S8)

where

$A=\frac{41\pi^{2}}{2048}\beta_{1}+\frac{\pi^{2}}{4}\beta_{2}+\frac{\pi^{2}}{32}\beta_{3}+\frac{\pi^{2}}{32}\gamma_{1}+\frac{9\pi^{2}}{64}\bar{\gamma}_{2}$ , (S9a)

$B=\frac{1}{4}\gamma_{1}$ , (S9b)

$C=\frac{189}{4096}\beta_{1}+\frac{1}{8}\beta_{2}+\frac{1}{16}\beta_{3}+\frac{23}{128}\gamma_{1}-\frac{1}{64}\bar{\gamma}_{2}$ , (S9c)

$D=\frac{95}{32768\pi^{2}}\beta_{1}+\frac{3}{64\pi^{2}}\beta_{2}+\frac{7}{512\pi^{2}}\beta_{3}+\frac{7}{512\pi^{2}}\gamma_{1}-\frac{13}{1024\pi^{2}}\bar{\gamma}_{2}$ . (S9d)

*γ_χ_* and *γ_ω_* are given by,

$\gamma_{\chi}=\frac{\pi(7\gamma_{1}+\bar{\gamma}_{2})}{105}\left(1+\frac{3h^{2}k_{m}^{2}}{2\pi^{2}}\right)-\frac{\pi^{3}(105\bar{\gamma}_{2}-19\gamma_{1})}{1120}\left(1-\frac{10(\gamma_{1}+3\bar{\gamma}_{2})h^{2}k_{m}^{2}}{(105\bar{\gamma}_{2}-19\gamma_{1})\pi^{2}}\right)\chi^{2}$ , (S10a)

$\gamma_{\omega}=\frac{16}{15}\left(\gamma_{1}\left(1-\frac{1}{2m^{2}}\right)-\frac{\bar{\gamma}_{2}}{112m^{2}}\right)\sim\frac{16}{15}\gamma_{1} \text{for} m^{2}>>1$ . (S10b)

In equation (S10a), *γ_χ_* was expanded to the second order of *χ* for its determination. Minimising Eq. (S5) by , we obtain,

. (S11)

Here we assume that *χ* is time-independent (), when the steady wave propagation is induced. Thus, from equations (S10a) and (S11), *χ* is obtained as,

$\chi=\frac{1}{\pi}\sqrt{\frac{16(7\gamma_{1}+\bar{\gamma}_{2})(2 \pi^{2}+3h^{2}k_{m}^{2})}{3\left((105\bar{\gamma}_{2}-19\gamma_{1})\pi^{2}-10(\gamma_{1}+3\bar{\gamma}_{2})h^{2}k_{m}^{2}\right)}}$ . (S12)

Minimising Eq. (S5) by *J_α_* and *J_β_*, and using the constraint of equation (9), we obtain,

, (S13a)

. (S13b)

Neglecting the fourth term in equation (S13a) under the assumption of , we obtain the solutions for these differential equations as,

, (S14a)

, (S14b)

where

$a\sim\frac{\rho_{e}E_{0}h}{M_{\alpha}}\frac{1}{1+\tau_{\alpha}^{2}\omega^{2}}$ , (S15a)

$b\sim\frac{\rho_{e}E_{0}h}{M_{\alpha}}\frac{\tau_{\alpha}\omega}{1+\tau_{\alpha}^{2}\omega^{2}}$ . (S15b)

$c\sim\frac{\rho_{e}E_{0}Z_{\alpha\beta}k_{m}\chi}{4Z_{\alpha}Z_{\beta}}\frac{(\tau_{\alpha}+\tau_{\beta})\tau_{\alpha}\tau_{\beta}\omega^{2}}{\left(1+\tau_{\alpha}^{2}\omega^{2}\right)\left(1+\tau_{\beta}^{2}\omega^{2}\right)}$ , (S15c)

$d\sim\frac{\rho_{e}E_{0}Z_{\alpha\beta}k_{m}\chi}{4Z_{\alpha}Z_{\beta}}\frac{\tau_{\alpha}\tau_{\beta}\omega(-1+\tau_{\alpha}\tau_{\beta}\omega^{2})}{\left(1+\tau_{\alpha}^{2}\omega^{2}\right)\left(1+\tau_{\beta}^{2}\omega^{2}\right)}$ . (S15d)

In equations (S15a)–(S15d) the relaxation times of *τ_α_* and *τ_β_* are given as,

$\tau_{\alpha}=\frac{Z_{\alpha}\xi h}{2M_{\alpha}}$, (S16a)

$\tau_{\beta}=\frac{2Z_{\beta}}{M_{\beta}k_{m}^{2}}$. (S16b)

Minimisation of equation (S5) by *J_γ_* and *V_c_* results in the following relations under the condition of :

, (S17a)

. (S17b)

Under the assumption that each impurity migrates much faster than LC molecule (), and that the propagation of the director field is much slower than the flow of LC (), the second terms in equations (S17a) and (S17b) are neglected. Using (S14b), (S17a) and (S17b), we obtain *V_c_* as

$V_{c}\sim\frac{\rho_{e}E_{0}Z_{\gamma c}h^{3}k_{m}}{16Z_{\gamma}\eta}\left(c(1+\cos2\omega t)+d\sin2\omega t\right)$. (S18)

is obtained by the minimisation of Eq. (S5) by :

. (S19)

Assuming that the wave frequency of *f_n_* is obtained by the time average of , and using equations (S15c), (S16b), (S18) and (S19), we obtain

, (S20)

where

$f_{n0}=\frac{\rho_{e}^{2}E_{0}^{2}Z_{\alpha\beta}Z_{\gamma c}\gamma_{\omega}\chi^{2}}{128\pi Z_{\alpha}M_{\beta}Z_{\gamma}\gamma_{1}}\frac{h^{3}k}{\eta}$ , (S21)

which are equations (19) and (21) in the main text, respectively.

Using equation (S8) and (S21), we obtain

$\frac{f_{n0}}{E_{0}^{2}}=\frac{Xhk}{1+Yh^{2}k^{2}}$ , (S22)

where

$X=\frac{\rho_{e}^{2}E_{0}^{2}Z_{\alpha\beta}Z_{\gamma c}\gamma_{\omega}\chi^{2}}{128\pi Z_{\alpha}M_{\beta}Z_{\gamma}\gamma_{1}}\frac{h^{2}}{A+Ch^{2}k_{m}^{2}+Dh^{4}k_{m}^{4}}$ , (S23a)

$Y=\frac{B}{A+Ch^{2}k_{m}^{2}+Dh^{4}k_{m}^{4}}$. (S23b)
